# Central Oregon obsidian from a submerged early Holocene archaeological site beneath Lake Huron

**DOI:** 10.1371/journal.pone.0250840

**Published:** 2021-05-19

**Authors:** John M. O’Shea, Ashley K. Lemke, Brendan S. Nash, Elisabeth P. Sonnenburg, Jeffery R. Ferguson, Alex J. Nyers, Danielle J. Riebe

**Affiliations:** 1 Museum of Anthropological Archaeology, University of Michigan, Ann Arbor, MI, United States of America; 2 Department of Sociology and Anthropology, University of Texas at Arlington, Arlington, TX, United States of America; 3 Lake Superior National Marine Conservation Area, Parks Canada, Canada; 4 University of Missouri Research Reactor Center (MURR), Columbia MO, United States of America; 5 Northwest Research Obsidian Studies Laboratory, Corvallis, OR, United States of America; 6 Department of Anthropology, University of Georgia, Athens, GA, United States of America; Max Planck Institute for the Science of Human History, GERMANY

## Abstract

Obsidian, originating from the Rocky Mountains and the West, was an exotic exchange commodity in Eastern North America that was often deposited in elaborate caches and burials associated with Middle Woodland era Hopewell and later complexes. In earlier times, obsidian is found only rarely. In this paper we report two obsidian flakes recovered from a now submerged paleolandscape beneath Lake Huron that are conclusively attributed to the Wagontire obsidian source in central Oregon; a distance of more than 4,000 km. These specimens, dating to ~ 9,000 BP, represent the earliest and most distant reported occurrence of obsidian in eastern North America.

## Introduction

Obsidian, or volcanic glass, is a prized raw material for knappers, both ancient and modern, with its lustrous appearance, predictable flaking, and resulting razor-sharp edges. As such, it was used and traded widely throughout much of human history. Since obsidian has unique, identifiable chemical signatures, it has also played an important role in the documentation and analysis of ancient exchange networks in places as diverse as the Arctic, the Eastern Mediterranean, Southeast Asia, and Mexico [1–6].

Within the continental United States and Canadian provinces, the principle sources of obsidian are found in the Pacific Northwest, as far east as South Dakota; and in the Southwest, particularly in Arizona and New Mexico [7–9]. While the use of obsidian is ubiquitous in the West, the pattern of archaeological occurrences East of the Rocky Mountains follows a distinct chronological pattern with obsidian appearing late as an important exotic good in the Middle Woodland Hopewell complex but only very sporadically before this [10,11]. Earlier occurrences are scattered across the Plains and further East but tend to be represented by very small numbers of flakes found within Late Archaic and Early Woodland contexts [12–16]. Additionally, most of this obsidian derives from the eastern most sources in Wyoming and New Mexico, along with a small amount from Idaho. Obsidian from the far western United States is rarely found in the East.

Given this established pattern of spatial and chronological distribution, the discovery of two obsidian flakes attributable to ~ 9000 yrs BP from the bottom of Lake Huron is unprecedented. The fact that both specimens are unambiguously derived from the Wagontire source in central Oregon, a distance of ~4,000 km, is extraordinary. These specimens represent the oldest and farthest east confirmed occurrence of western obsidian in the continental United States. In this report we document the context of the find, describe the specimens, and present the detailed compositional data that allows the specimens to be linked to their central Oregon source.

## Materials and methods

**Fig 1:**
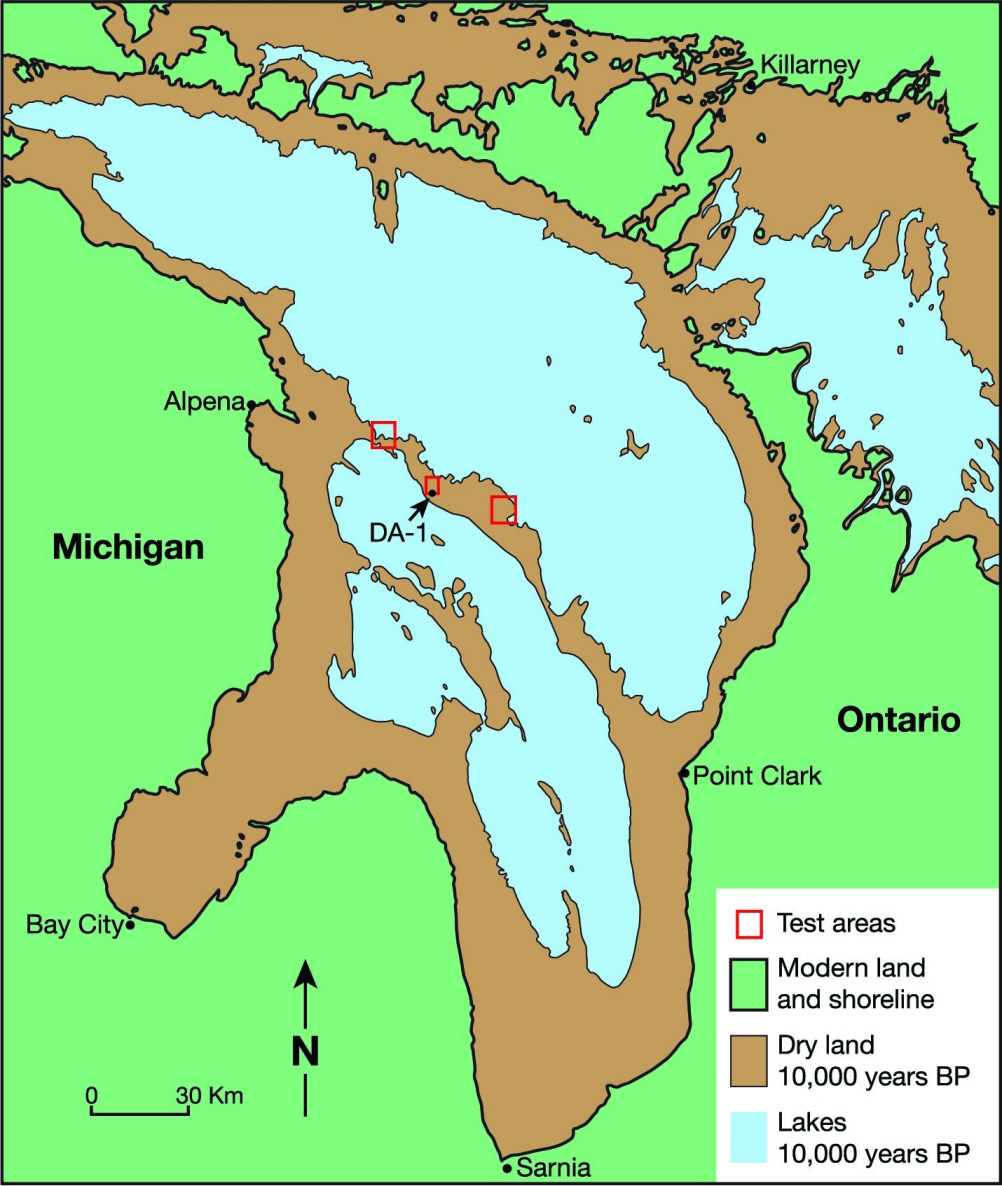
Map of the Lake Huron Basin. Areas shaded green represent the modern coast and land surface; brown areas represent dry land during the Lake Stanley lowstand, and blue represents the location of the lakes at approximately 10,000 yrs BP. Red rectangles represent areas where archaeological research has been conducted. The location of the obsidian finds, sample DA-1, is indicated.

Over the past 18,000 years water levels in the North American Great Lakes have fluctuated significantly. These dynamic water levels, coupled with isostatic rebound since the last ice age, exposed and then submerged vast areas of land [17]. At the lowest water level, these areas were inhabited by plants, animals, and humans [18,19]–a now submerged paleolandscape which holds evidence of a past occupation. During the Lake Stanley low water stage, between 10,000 and 8,000 BP [20,21], the Alpena-Amberley Ridge (AAR) landform represented a dry land corridor that linked northeastern Lower Michigan with southern Ontario (Fig 1). With the rise in water levels at the end of Lake Stanley, the landform was inundated and never again exposed. As such it provides a unique record of final Pleistocene and Early Holocene environments and cultural adaptations in the Great Lakes region. Archaeological research on this landform has documented constructed features including stone drive lines, hunting blinds, and fire rings [22,23]. Excavations at archaeological sites on the AAR have produced lithic debris and debitage [19].

The obsidian artifacts were recovered from sample DA-1 comprised of 3 litres of sediment that was hand excavated at a depth of 105’ ft (32 m) in an area between two identified hunting structures, TV-1 and V-Cluster [22]. Radiocarbon determinations associated with this depth/elevation date the occupation between 9000 and 8800 cal yr BP [19,21] (S1 Table). Paleoenvironmental indicators, specifically testate amoebae and air breathing snails, indicate that the immediate area was a swamp/bog with scattered trees in a location generally characterized as subarctic [24].

**Fig 2:**
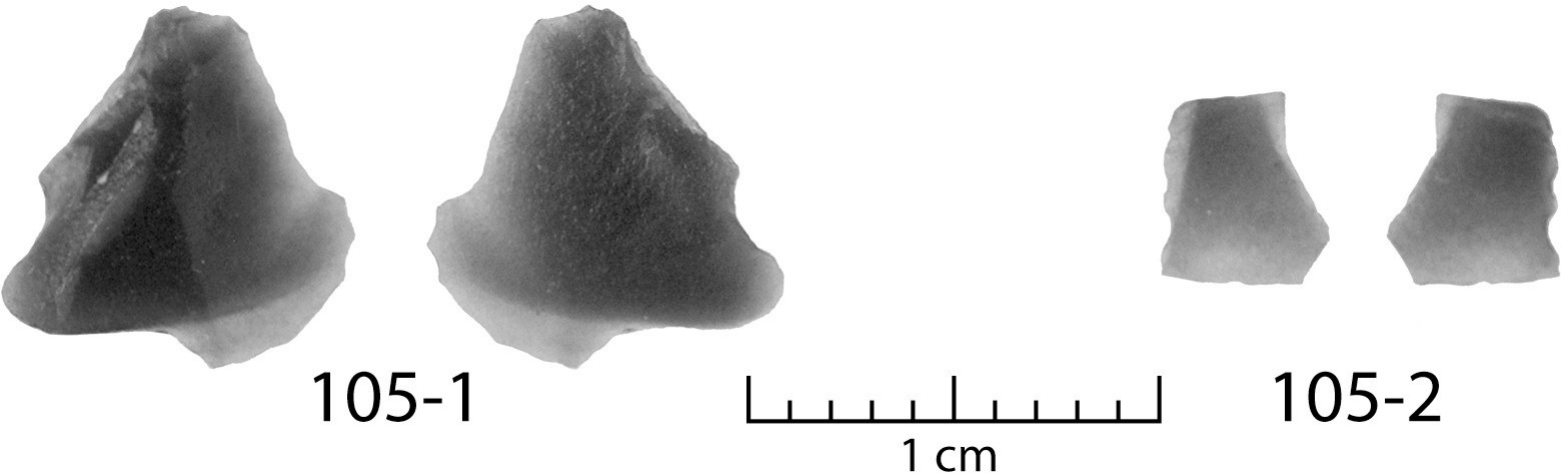
Photographs showing the front and back of the two obsidian flakes. Scale is in centimeters.

**Fig 3:**
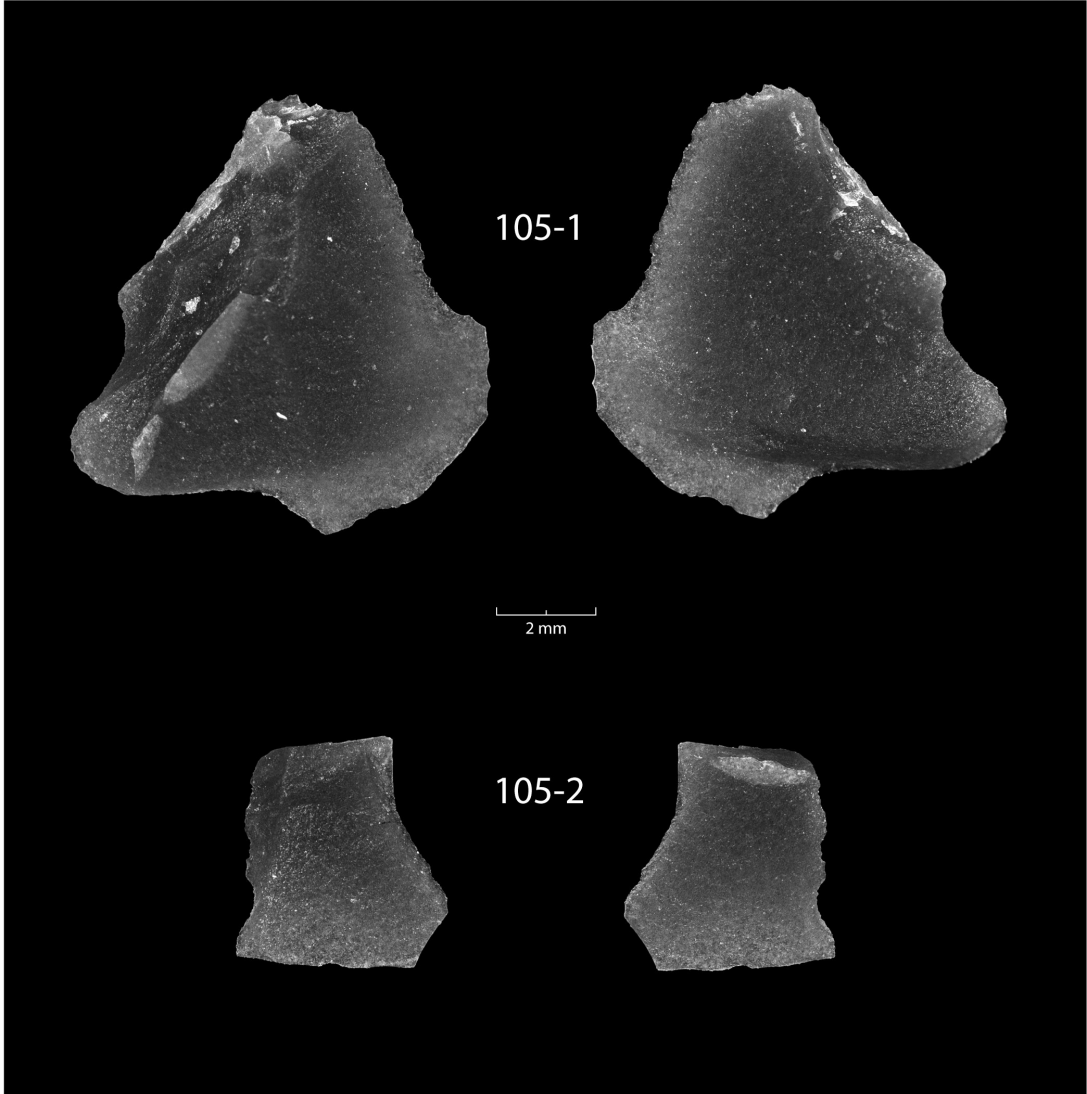
Photomicrographs of the two obsidian flakes on a dark field to highlight flake modification. Scale is in millimeters.

Two small flakes were recovered from sample DA-1 (Table 1, Figs 2 and 3). Both are curated in the Great Lakes Range of the Museum of Anthropological Archaeology at the University of Michigan. The larger specimen (laboratory number 105–1) is a mostly complete, roughly triangular, biface thinning flake made from a black and translucent material with a sub-vitreous texture. The flake has clear ventral and dorsal faces with evidence of flake scars from an earlier stage of reduction on the latter. One lateral edge leading to a crushed platform is feathered in termination, while the other is step fractured, possibly from two or three failed percussion flake removal strikes, or perhaps from use. Opposite of the platform, the third edge terminates in an irregular fashion. Half of the edge plunges in a way to create a rolled termination that would have left a hinge or step on the biface, while the other half of this edge feathers out over top of the plunge in a way unique to obsidian.

**Table 1:** Sample DA-1 specimen metrics.

Specimen	Length (mm)	Width (mm)	Thickness (mm)	Weight (g)
105–1	7.3	6.5	1.6	0.07
105–2	2.3	2.9	0.4	0.01

The second specimen (laboratory number 105–2) is a small, very thin, translucent flake on a material visually similar to the larger specimen. The morphology is consistent with light percussion flaking on a biface. The flake has a ventral and dorsal face, but no platform. It is likely that this flake detached along with another flake during bifacial refurbishment.

The small size and technological character of the obsidian flakes suggest they represent a fairly delicate biface refurbishment episode. The flakes do not display obvious evidence of use as tools themselves. Given their spatial association, raw material, and technological character, it is likely that these two flakes are from the same bifacial tool and were produced as a byproduct of a single resharpening event.

Lithic materials recovered from the Lake Huron archaeological sites are typically fashioned on chert cobbles collected from local glacial deposits [19]. The two specimens from DA-1 stood out as unique given their qualitative characteristics and were identified as likely obsidian. To test this, the specimens were sent to the *Field Museum* and analyzed using a Bruker TRAcER III-SD portable XRF apparatus, and the identification of obsidian was confirmed. The North American geological database at the *Field Museum* was not robust enough to identify the obsidian source for the specimens so the samples were transferred to the *Northwest Research Obsidian Studies Laboratory*. There the samples were analyzed with another XFR, confirmed again as obsidian with a likely source area of Central Oregon.

Given the remarkable distance between the find spot and the apparent source as well as the limitations of XRF analysis, the samples were further analyzed with a complementary method, neutron activation analysis (NAA). As the Lake Huron samples were small and potentially unique, the excavators were unwilling to undertake destructive analysis. As such the samples were transferred to the *University of Missouri Research Reactor center (MURR)* where analysis was conducted using only short irradiation NAA. This method allowed comparison with the laboratory’s global database of sources and focused on an additional set of elements not accessible to XRF. More specific information on the materials and methods of underwater sample recovery and processing as well as NAA procedures can be found in S1 Text.

## Results

**Fig 4:**
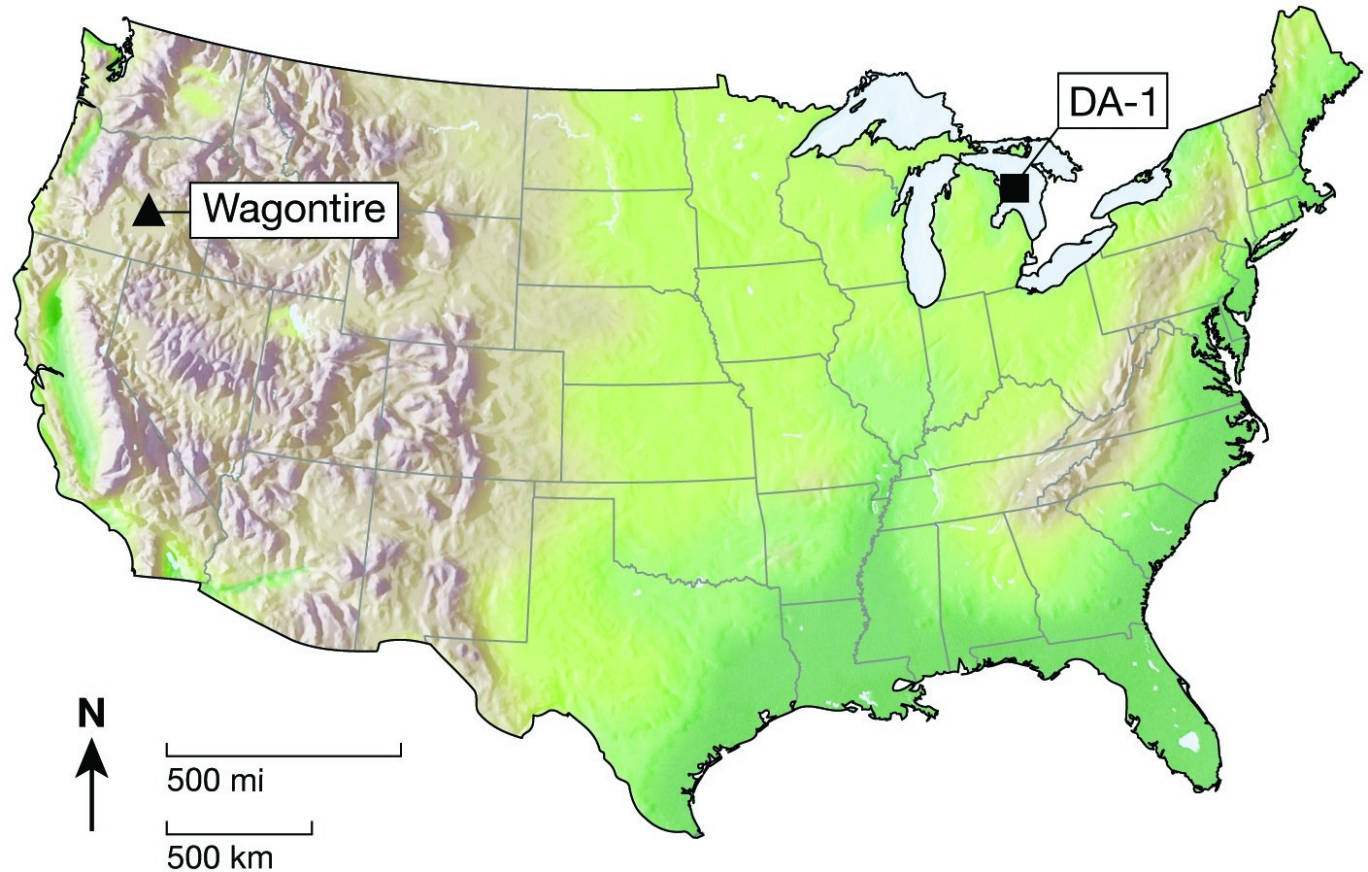
Map of the continental United States, with the location of the Wagontire obsidian source (triangle) in central Oregon and the DA-1 obsidian find spot (square) in Lake Huron. The straight line distance between these points is over 4,000 km.

**Fig 5:**
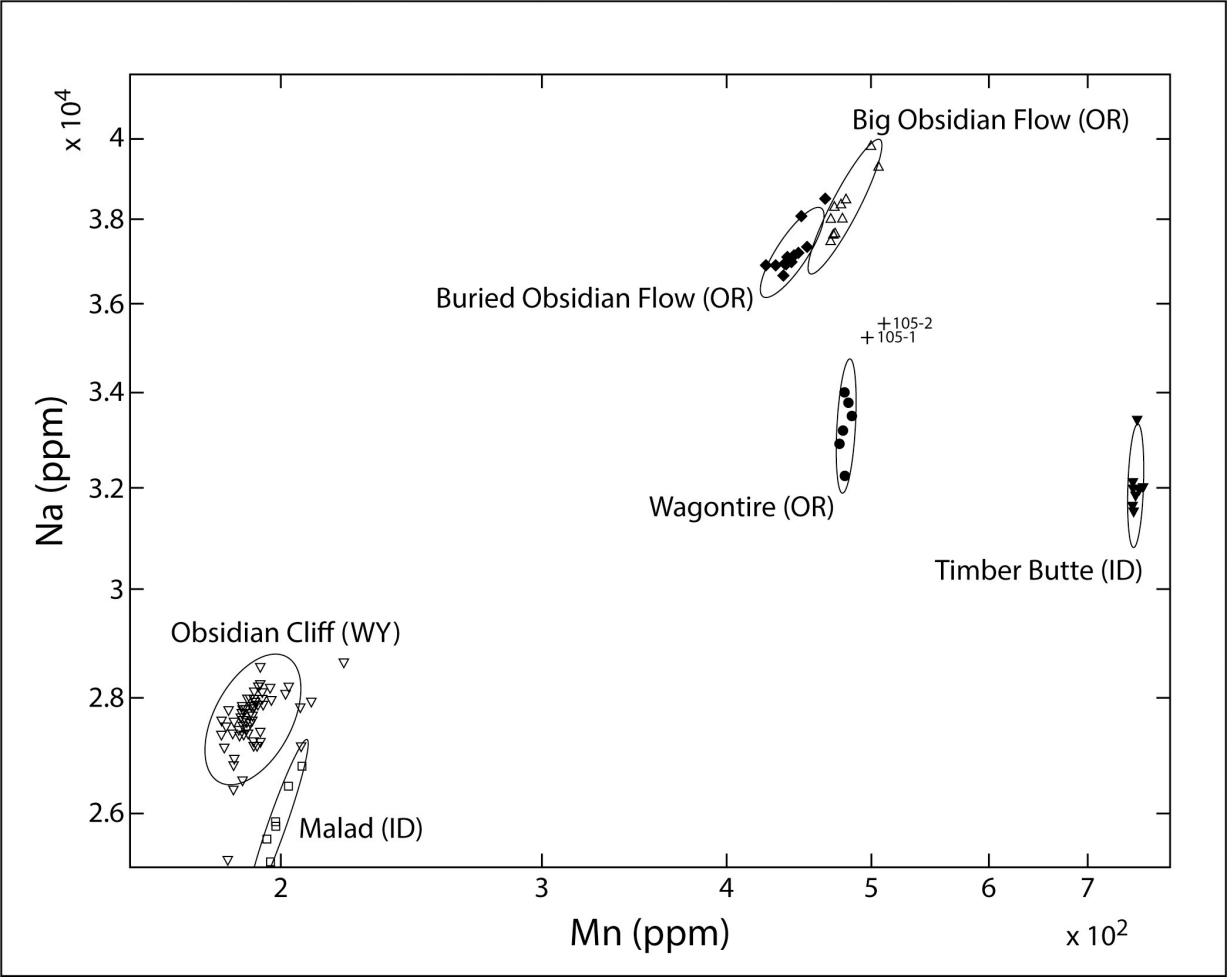
Displays measurement for two elements, sodium and manganese for several commonly utilized obsidian sources as well as the two Lake Huron artifacts. Ellipses represent statistical confidence intervals for geological source materials. It is clear that the Lake Huron artifacts most closely match the geological signature generated for the Wagontire source.

The results of analysis using energy dispersive x-ray fluorescence (EDXRF) at the *Northwest Research Obsidian Studies Laboratory* are presented in Table 2A. The diagnostic trace element values used to characterize the samples were compared to those for known obsidian and fine-grained volcanic sources reported in the literature and with unpublished trace element data collected through analysis of geological source samples [25]. Based on these comparisons, the two Lake Huron specimens were traced to source areas in central Oregon; nearly 4,000 km away (Fig 4). Three source areas in Central Oregon (Wagontire, Big Obsidian Flow, and Buried Obsidian Flow) were very similar in all of the elements typically analyzed by XRF but the most obvious difference was barium. Barium is somewhat difficult to measure by XRF, especially in smaller artifacts, therefore NAA analysis was used to differentiate between the central Oregon sources. Table 2B presents the results of the Short NAA run. The barium in the Obsidian Flow sources is about 900 ppm, but it is 1500 ppm in Wagontire. The two Lake Huron artifacts are both right at the expected 1500 ppm for Wagontire. There are similar differences for sodium with a similar match for Wagontire (Fig 5). The NAA data reveal that the two Lake Huron flakes are a clear match with the Wagontire source.

**Table 2:** Elemental concentrations for ED-XRF and short-irradiation NAA of Wagontire source samples and artifacts. a. Results of XRF Analysis. b. Results of Short Run NAA Analysis.

**ED-XRF Data (ppm)**	**RB**	**SR**	**Y**	**Zr**	**Nb**	**Ba**
Wagontire Mean Values (n = 61)	107	43	55	330	21	1441
Wagontire Standard Deviations (n = 61)	4	2	2	3	2	70
Specimen 105–1	115	48	52	360	25	942
Specimen 105–2	102	46	46	297	19	NM
**NAA Short Irradiation Data (ppm)**	**Na**	**Al**	**K**	**Mn**	**Ba**	**Dy**
Wagontire Mean Values (n = 6)	33286	71001	35924	480.2	1425	9.12
Wagontire Standard Deviations (n = 6)	644	2436	1214	3.2	27	0.29
Specimen 105–1	35230	72735	35515	497.4	1545.1	8.91
Specimen 105–2	35522	36622	36622	510.0	1508.2	9.13

All trace element values reported in parts per million.

NM = Not measured.

The NAA results were compared to the entire database of sources at MURR, including almost every known source worldwide. The only possible match was Wagontire. As such, the identification of both artifacts as obsidian was independently confirmed by three laboratories and two methods, while the specific sourcing of the artifacts to Central Oregon was confirmed by laboratories using both high definition XRF and NAA, and the specific source, Wagontire, was determined by NAA and comprehensive comparison to global obsidian sources.

## Discussion

The Lake Huron finds represent both the earliest and most easterly occurrence of western obsidian in the continental United States that have been recovered from a secure archaeological context. It is worth noting in this regard that this is the first documented archaeological context for Wagontire obsidian outside the state of Oregon. It is likely these artifacts were deposited as the result of a single event of biface refurbishment, suggesting a high level of curation. Yet, while clearly exotic, it is noteworthy that they are attributable to a relatively routine activity in a kill area and do not occur as part of any special or ritual context. The extreme distance between source and find location raises renewed questions concerning the scale of mobility and social connectedness during the Pleistocene-Holocene transition.

While it is notoriously difficult to parse out the specific means for the long-distance movement of goods among mobile foraging populations (e.g. direct procurement, embedded procurement, down the line exchange, etc.), it seems probable that these specimens arrived in the Great Lakes through multiple hands, rather than through any direct access to the central Oregon obsidian source.

The artifacts reported therefore likely reflect the existence of continent-wide social networks that extended west-to-east across the recently deglaciated landscapes of North America at the end of the Pleistocene. In other geographic areas, e.g. Alaska, it has been noted that recently deglaciated landscapes are productive new lands which were often exploited by small, highly mobile bands utilizing a miniaturized lithic technology with micro-blades [26–31]. While lithic miniaturization is a global phenomenon in the Early Holocene [32–36], the North American cases, including this new find from Lake Huron, seem particularly suited to the habitation of new lands exposed during glacial retreat. It is tempting to view the long-distance west-to-east movement of obsidian reported here as being channeled along these recently deglaciated landscapes.

Given the evidence at hand, we cannot determine whether the obsidian came to the Great Lakes as a chance event or was part of a more frequent and regular occurrence. While the finds likely reflect the existence of an extensive network of contacts along recently deglaciated landscapes at the end of the Pleistocene, it is impossible to know whether the Lake Huron peoples actually knew where the exotic stone came from. What we can say with confidence is that there is no plausible natural process that can account for the presence of the obsidian flakes in this location.

The Lake Huron finds fit within the broader, global context of obsidian movement generally, and the long-distance movement of raw materials in Late Pleistocene North America more specifically. Table 3A provides a global sample of long-distance movement of obsidian. Significantly, the longest reported distances of movement involve the use of boats in Melanesia and Northeast Asia, suggesting that boat travel and transport aids in the long-distance movement of goods.

**Table 3:** Examples of raw material transport distances. a. Obsidian Transport. b. Paleoindian Long-Distance Movement.

**Geographic Area**	**Distance (km)**	**Reference(s)**
Melanesia and New Zealand	2000 +	[37,38]
Mexico–Oklahoma	1800	[39]
Northeast Siberia	1300	[40]
Idaho–Minnesota	1000	[41]
Northeast Asia/Japan	800–1000	[3]
Patagonia	300–1000+	[7]
**Raw Material**	**Distance (km)**	**Reference(s)**
Knife River Flint	1500	[42]
Edwards Plateau Chert	955	[43,44]
Wyandotte Chert	600	[45,46]
Alibates Dolomite	584	[44]
Munsungun Chert	400	[47]

The Lake Huron specimens date to the Late Pleistocene/Early Holocene in North America, a time of Paleoindian occupation–an archaeological culture which is known for the long-distance movement of high-quality tool stone [37,38,45]. While obsidian is noted in some assemblages, the raw materials that traveled the farthest distances are flint and chert, with some items moving upwards of 1500 km (Table 3B). These examples of long-distance movement of both obsidian generally, and flint more locally demonstrate that the Lake Huron samples are some of the longest distances recorded while also fitting within the context of hunter-gatherer movement and raw material transport.

Overall, the recovery of these specimens from beneath Lake Huron is significant, as it represents a secure archaeological context and underscores the remarkable potential of underwater investigations. It is likely that additional research underwater, as well as targeted research on land, will further elucidate the character of Terminal Pleistocene/Early Holocene exchange networks. Regardless of the flakes’ use and the frequency of these interactions, the materials themselves represent trans-continental connections spanning from the West Coast to the Great Lakes. These specimens provide greater resolution, as well as greater complexity, to an important and poorly understood time period in the North American past.

## Supporting information

S1 Table 14C dates associated with the human occupation on the AAR. (DOCX)

S1 Text Sample recovery and processing, NAA procedures. (DOCX)
