# Supplementary material for: Central Oregon obsidian from a submerged early Holocene archaeological site beneath Lake Huron
Source: PLoS One. 2021 May 19;16(5):e0250840. doi: 10.1371/journal.pone.0250840 (PMC8133412; doi:10.1371/journal.pone.0250840)
Supplement: S1 Table — (DOCX) [file pone.0250840.s001.docx]

Table S1. 14C Dates Associated with the Human Occupation on the AAR.

| Laboratory | Designation | Material | Age bp | Age Err | d13C | 2Sigma Cal Range BP* | Median |
| --- | --- | --- | --- | --- | --- | --- | --- |
| Arizona | AA95226 | wood | 8038 | 46 | -25.5 | 9073-8723 | 8904 |
| NOSAMS | 109842 | wood | 7960 | 55 | -25.12 | 8995-8641 | 8828 |
| Georgia | 19168 | Wood | 8102 | 26 | -25.6 | 9113-8995 | 9023 |
| NOSAMS | 110722 | charcoal | 8080 | 35 | -26.54 | 9125-8789 | 9012 |
| Georgia | 19163 | wood | 8432 | 27 | -26.3 | 9519-9429 | 9469 |
| Georgia | 19170A | wood | 8163 | 26 | -26.2 | 9246-9015 | 9086 |
| Georgia | 19170B | charcoal | 8797 | 27 | -27.0 | 10111-9691 | 9817 |
| Georgia | A22925 | wood | 8201 | 29 | -28.1 | 9267-9032 | 9162 |

* Calibration based on Oxcal 4.3
