# Supplementary material for: Central Oregon obsidian from a submerged early Holocene archaeological site beneath Lake Huron
Source: PLoS One. 2021 May 19;16(5):e0250840. doi: 10.1371/journal.pone.0250840 (PMC8133412; doi:10.1371/journal.pone.0250840)
Supplement: S1 Text — (DOCX) [file pone.0250840.s002.docx]

Sample Recovery and Processing

AAR sites are discovered and documented via a multilayered search strategy [46]. An initial survey was conducted using side-scan sonar within two pilot search areas, one a square 7.5 km to a side and the second a square of 4.5 km per side. Subsequently, a partially overlapping area of 115 km^2^ was mapped using multibeam sonar. Side-scan survey was conducted using a digital side-scan sonar unit (Imagenex), at a frequency of 330 kHz and a depth of 20 m, mapping overlapping swaths of 200m. Multibeam survey was conducted using an R2Sonic 2024 multibeam echo sounder with an F180 vessel attitude and position system. Finer resolution survey was conducted using an Oceanserver *Iver 3* autonomous underwater vehicle (AUV), using 50 m swaths at 5 meters above the bottom. Targets of interest were examined using an Outland 1000 remote operated vehicle (ROV), equipped with UWL-500 LED lights, UWC-360D dual high-definition video cameras (color and black and white), and a manipulator. The ROV also carried a Tritech MicroNav100 tracking sonar to allow its location to be recorded in real time.

The specimens described in this paper were recovered from manual excavation conducted by scuba-trained archaeologists. The DA-1 sample contained 3 liters of sediment. The sample was screened and sorted for lithic artifacts and larger organics in the laboratory using a low powered microscope.

Neutron Activation Analysis Procedures

NAA of ceramics requires removal of a portion of an artifact, physical removal of the exterior surface, and homogenization of the sample by grinding into a fine powder resulting in a clearly destructive process [47]. Obsidian analysis does not require grinding due to obsidian’s naturally homogenous structure, but standard NAA of obsidian renders the pieces (either artifacts or geologic samples) radioactive and unsafe to return to curation facilities. Thus, the technique is considered destructive even though the artifact is not actually consumed during analysis. NAA at MURR actually consists of two separate irradiations: long and short. The long irradiation sample is measurably radioactive for many thousands of years, but the short irradiation process imparts such a low dose of radiation onto the sample, that it is safe to handle the artifacts after just a couple months.

The short irradiation method [48] utilizes small high-density polyethelene vials, and if the specimens are small enough to fit into the vials then they can be non-destructively analyzed by short-NAA. Given the rarity of the two artifacts in this study, destruction was not a reasonable option. This approach has been used successfully in cases of small artifacts requiring more elemental data than ED-XRF alone can provide [e.g. 49]. ED-XRF has limitations with smaller artifact sizes [50] and these are precisely the artifacts ideal for non-destructive short NAA.

Individual sample weights were recorded to the nearest 0.01mg using an analytical balance. Along with the unknown samples, standards made from National Institute of Standards and Technology (NIST) certified standard reference material of SRM-278 (obsidian rock) and quality controls included samples of SRM-1633b (coal fly ash) and the Alca obsidian source in Peru. Concentrations are determined for Al, Ba, Cl, Dy, K, Mn, and Na. NAA methods are discussed in detail by Glascock [47-48].

**References**

46. O’Shea J, Lemke A. A layered approach for the discovery and mapping of prehistoric sites beneath Lake Huron. Marine Technol Soc J. 2020;54(3):23-32.

47. Glascock M. Characterization of Archaeological Ceramics at MURR by Neutron activation Analysis and Multivariate Statistics. In Neff H, editor. Chemical Characterization of Ceramic Pastes in Archaeology. Madison: Prehistory Press, 1992. p. 11-26.

48. Glascock M, Neff H, Stryker K, Johnson T. Sourcing archaeological obsidian by an abbreviated NAA procedure. J Radioanal Nucl Ch. 1994;180(1):29-35.

49. Izuho M, Ferguson J, Glascock M, Oda M, Akai F, Nakazawa Y, Sato H. Integration of Obsidian Compositional Studies and Lithic reduction Sequence Analysis at the Upper Paleolithic Site of Ogachi-Kato 2, Hokkaido, Japan. In: Ono A, Glascock M, Kuzmin YV, Suda Y, editors. Methodological Issues for Characterisation and Provenance Studies of Obsidian in Northeast Asia. Oxford:BAR S2620; 2014. P. 125-142.

50. Ferguson J. Fluorescence of Obsidian: Approaches to Calibration and the Analysis of Small Samples. In: Shugar A, Mass A, editors. Handheld XRF for Art and Archaeology Leuven: Leuven University Press; 2012. p. 400-421.
